# Flow Cytometric Analysis of CD133- and EpCAM-Positive Cells in the Peripheral Blood of Patients with Lung Cancer

**DOI:** 10.1007/s00005-013-0250-1

**Published:** 2013-08-20

**Authors:** Tomasz Skirecki, Grażyna Hoser, Jerzy Kawiak, Dariusz Dziedzic, Joanna Domagała-Kulawik

**Affiliations:** 1 Laboratory of Flow Cytometry, Medical Center of Postgraduate Education, Warsaw, Poland; 2 Department of Pneumonology and Allergology, Medical University of Warsaw, Banacha 1a, 02-097 Warsaw, Poland; 3 Department of Surgery, National Institute of Tuberculosis and Lung Diseases, Warsaw, Poland; 4 Institute of Biocybernetics and Biomedical Engineering, Polish Academy of Science, Warsaw, Poland

**Keywords:** Lung cancer, Cancer stem cell (CSC), CD133, EpCAM, Metastases

## Abstract

Lung tumors are characterized by their high metastatic potential, which is the main cause of therapeutic failure. However, the exact cellular origin of metastasis remains unknown. Since the introduction of the cancer stem cell theory, lung cancer stem cells (LCSCs) have been thought to represent metastasis-founding cells. The current study aimed to evaluate whether LCSCs could be found in the circulation. Expression of the stem cell markers CD133 and EpCAM was confirmed in tumor and normal lung tissue by flow cytometry. Then, this technique was further used to investigate the expression of CD133 and EpCAM in the peripheral blood of 41 patients with primary lung cancer. Putative LCSCs (CD133^+^EpCAM^+^) were present in 6/7 tumor samples, and CD133^+^EpCAM^+^ cells were identified in the blood samples of 15 patients at a median level of 40/ml of blood. EpCAM^+^ cells were detected in 60 % of the patients, and the number of these cells was higher in patients with adenocarcinoma than patients with squamous cell carcinoma and was also higher in patients with less advanced disease. Moreover, the frequency of this subpopulation significantly correlated with the circulating level of SSEA-4^+^ cells. Additionally, CD133^+^EpCAM^−^ cells were found in 87 % of the patients, and the numbers of these cells were significantly higher in patients with distant metastases and correlated with disease stage. This study confirmed the presence of an LCSC subpopulation with a CD133^+^EpCAM^+^ phenotype in the tumors and blood of patients with lung cancer, and these results suggest an important role for CD133 and EpCAM in lung cancer progression and their potential application as novel biomarkers of the disease.

## Introduction

Lung cancer is an aggressive, malignant neoplasm with approximately 1.3 million new cases worldwide each year, and poor prognoses lead to high levels of mortality among men and women. However, 5-year survival rates greater than 15 % are now being reported (Jemal et al. 2010). Two main histological types of lung cancer can be identified: non-small cell lung carcinoma (NSCLC) and small cell lung carcinoma (SCLC), which is highly aggressive and responsive to chemotherapy. Adenocarcinoma, an NSCLC subtype, was recently shown to be the most frequently observed type of lung cancer and is a candidate for targeted therapy (Alberg et al. 2007). The low 5-year survival rate for patients with lung cancer is related to the occurrence of early, widespread metastases (including those to the bone marrow, bones, brain, liver, and pleural cavity) and a high rate of relapse, regardless of whether initial treatment led to remission (Krzakowski 2010).

Since the introduction of the cancer stem cell (CSC) hypothesis in relation to acute myelogenous leukemia (Lapidot et al. 1994), there is a growing body of evidence to suggest that cancer development and resistance are driven by a rare subpopulation of cells with the ability to self-renew and generate the broad heterogeneity of the primary tumor mass following xenotransplantation into immunodeficient mice (Clevers 2011). Such CSCs have been found and characterized in many solid tumors, including breast, brain, colon, pancreatic and lung tumors (Al-Hajj et al. 2003; Eramo et al. 2008; Li et al. 2007; Ricci-Vitiani et al. 2007; Singh et al. 2003). Lung cancer stem cells (LCSCs) were originally isolated using the CD133 antigen (prominin-1), which is a common stem cell marker with an unknown biological function. Lung cancer cells expressing CD133 were found to be more resistant to chemotherapeutics and form tumors when injected into SCID mice in low numbers (Eramo et al. 2008). Other studies revealed that these cells express the embryonic transcription factor Oct-3/4, which is essential for maintenance of unique stem-like properties (Chen et al. 2008). Another stem cell marker is stage-specific embryonic antigen-4 (SSEA-4), which is an embryonic stem cell surface protein that is lost during differentiation (Draper et al. 2002).

Metastases originate from circulating tumor cells (CTCs) that migrate through the blood and lymph. Circulating lung cancer cells have been detected using various methods, such as polymerase chain reaction (PCR) and immunocytochemistry (Tsavellas et al. 2002). Moreover, lung cancer cells have been identified in the blood based on their expression of cytokeratin or epithelial cell adhesion molecule (EpCAM/CD326) (Krebs et al. 2011). More recently, circulating cancer cells were shown to express a chemokine receptor for stromal derived factor-1 (SDF-1), which is the CXCR4 receptor. It has also been suggested that cells expressing CXCR4 may undergo chemotaxis toward gradients of SDF-1 and therefore anchor at sites rich in SDF-1 (e.g., bone marrow) to form metastases (Reckamp et al. 2009). Although most cancer cells express CXCR4, not all are able to form tumors (Eramo et al. 2008), and it is likely that not all of these cells are capable of forming metastases.

Taken together, these observations encouraged us to investigate whether cells with an LCSC phenotype could be found in the peripheral blood of patients with lung cancer. We first confirmed the presence of CD133^+^EpCAM^+^ cells in freshly obtained tumor tissue and then sought to identify cells of this phenotype in the peripheral blood of patients with lung cancer. We also aimed to determine whether the identification of circulating stem cells could be correlated with various clinical data and patient disease stage. For these purposes, flow cytometry and staining using monoclonal antibodies targeting the cell-surface expression of EpCAM and CD133 were used.

## Materials and Methods

### Patients

Tumor samples were obtained from patients with lung cancer who had undergone elective tumor surgical resection (*N* = 7). Normal lung tissue specimens were obtained from the same patients. These sections were resected from macroscopically healthy lung parenchyma in the marginal zone. All patients were primary cancer patients and received no anticancer treatment prior to surgery.

Blood samples were obtained from patients who were admitted to the Department of Pneumonology of the Medical University of Warsaw and who had been diagnosed with lung cancer (*N* = 41). These patients did not receive anticancer treatment prior to blood sample collection. Blood was also collected from patients in addition to those who provided tumor samples.

Samples of peripheral blood were also collected from 15 healthy volunteers who served as the control group.

Only patients with histologically/cytologically confirmed lung cancers were included in the study group. Demographic data and data regarding the clinical stage of the disease were collected. The actual TNM classification of disease stage was used (Goldstraw 2009). All patients underwent thoracic computed tomography (CT) scan, bronchoscopy, and ultrasonography of the abdominal organs. Lymph node involvement was evaluated based on the CT scan results and, if indicated, endobronchial ultrasound-guided transbronchial needle aspiration (EBUS/TBNA). Distal metastases were verified in symptomatic patients by appropriate imaging methods. The patient characteristics are described in Table 1. Most patients had advanced-stage disease (60 % in stage IV) and half of the patents had distant metastases at the time of diagnosis.

**Table 1:** Clinical characteristics of patients with lung cancer whose blood was analyzed for circulating cancer stem cells

N	41
Mean age (years)	67 (49–92)
Male/female	27/14
Smoking history (pack years)	36 ± 18 all patients were smokers or ex-smokers
NSCLC	34
Squamous cell carcinoma	9
Adenocarcinoma	8
Non other specified non-small cell carcinoma	17
SCLC	7
Stage: IB/II/IIIA/IIIB/IV	1/4/4/6/23
Lymph nodes+	28 (68 %)
Distal metastases+	23 (56 %)

*NSCLC* non-small cell lung cancer, *SCLC* small cell lung cancer

Patients provided informed consent to be included in this study, which had been approved by the local ethics committee.

### Flow Cytometry

Due to our preliminary results (unpublished data) which showed that EpCAM-positive cells could be found in the peripheral blood of cancer patients, we decided not to use any pre-enrichment steps which can produce bias by loss of particular cells. One milliliter of peripheral blood was obtained. A total of 50 μl of blood was stained using the following murine antibodies: APC-conjugated anti-CD133 (Miltenyi Biotec, Bergisch Gladbach, Germany), FITC-conjugated anti-EpCAM, and PE-conjugated anti-SSEA4 (Beckton Dickinson, San Jose, CA, USA). Isotype control antibodies (BD Sciences, San Jose, CA, USA) were used for control staining. After 30 min of incubation at 20 °C, erythrocytes were lysed with BD lysing solution and washed with 2 % newborn calf serum in physiological buffer solutions (PBS). The cells were subsequently fixed in 0.5 % paraformaldehyde.

Tumor specimens and normal lung tissues were placed in 0.9 % NaCl and immediately transported to the laboratory. Samples were digested by incubation in 0.1 % type I collagenase (Sigma Aldrich, USA) in PBS for 2 h at 37 °C and then filtered through 40-μm steel mesh. The cell suspensions were then washed with PBS and resuspended in PBS for staining. The antibody staining was performed in the same manner as for the blood cells.

**Fig. 1:**
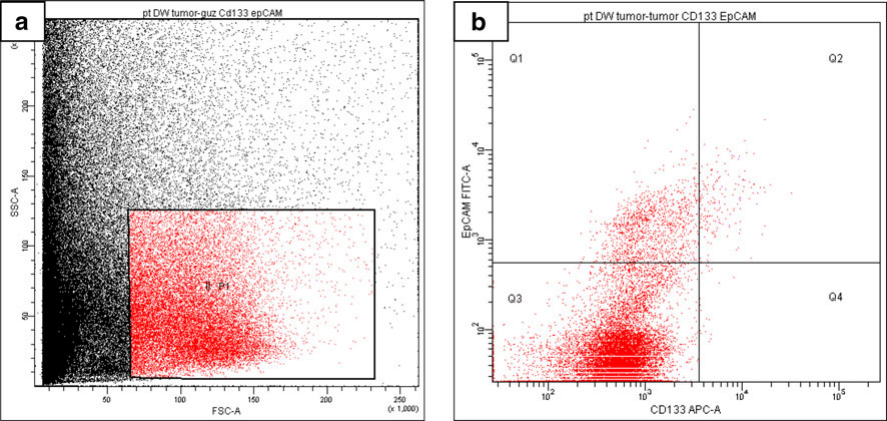
An example of dot plots of cells obtained from a digested lung tumor that was stained for CD133 and EpCAM expression. **a** Morphological cytogram showing the gating strategy for the dispersed tumor cells. **b** Of the gated cancer cells, 5.6 % expressed EpCAM only (Q1) and 0.82 % were positive for both EpCAM and CD133 (Q2)

**Fig. 2:**
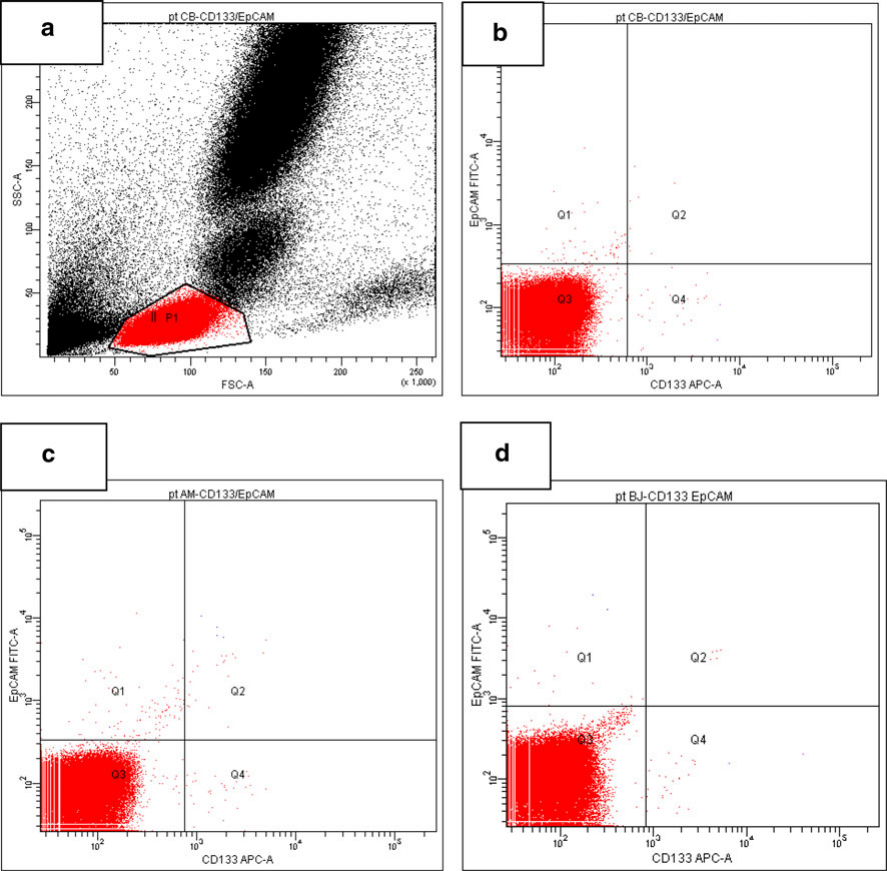
Dot plots for the representative peripheral blood immunocytochemical analysis. **a** The expression of stem cell markers was analyzed in the lymphocyte gate. **b** The blood from this patient contained 0.0119 % EpCAM^+^ cells (Q1), 0.0059 % CD133^+^ cells (Q4) and 0.0016 % CD133^+^EpCAM^+^ cells (Q2). Plots **c**, **d** show the results from two other patients who had 0.0018 and 0.0019 % CD133^+^EpCAM^+^ cells (Q2)

Antibody-stained samples were analyzed using a FACSCanto II flow cytometer and FACSDiva software (Becton-Dickinson, San Jose, CA, USA). As CTC are expected to be very rare, we collected at least 50,000 mononuclear cells from each sample. The percentage of positive cells was recorded, and the absolute number of positive cells per 1 ml of blood was calculated using the total white blood count. Events were collected from the lymphocyte gate on the FSC/SSC dot plot and read on a CD133^+^ versus EpCAM^+^ dot plot. The gating strategy and staining of a probe is presented in Figs. 1 and 2. Efforts were made to analyze 500,000–1,000,000 events from each sample. The flow cytometry blood cell analysis results are presented as the proportion of cells, the value of which is independent of the sample size, and as the absolute number of cells in 1 ml of blood (as a derivative of white blood count).

### Statistical Methods

Comparisons between two groups were performed using the Whitney–Mann *U* test. Differences were considered significant for *p* < 0.05. The results are expressed as the median and *P*_25_–*P*_75_ values. The relationships between the data were examined using the Spearman’s rank correlation coefficient. Correlations with *R* ≥ 0.4 and *p* < 0.05 were considered significant. Calculations were performed using Statistica 9.0 software.

## Results

### Tumor Tissue

The median patient age was 60 years, and six out of seven patients were male. Four of the tissue samples were from cases of adenocarcinoma and three from cases of squamous cell carcinoma. CD133^+^ and EpCAM^+^ cells were present at a higher number in all of the analyzed tumors in comparison to normal lung tissues (Table 2). The differences between normal and malignant tissues were not significant, which was likely due to the few number of samples examined (*N* = 7). The rare double-positive CD133^+^EpCAM^+^ cell subset was also present in six of the seven tumor samples at a 2.7 times higher proportion than in the healthy lung tissue [median values 0.1 (0.01–0.39 %) and 0.037 (0.01–0.0443 %), respectively; *p* = 0.16].

**Table 2:** Proportion of CD133^+^ and EpCAM^+^ cells in lung cancer and normal lung tissues

	CD133^+^EpCAM^−^ (%)	CD133^+^EpCAM^+^ (%)	CD133^−^EpCAM^+^ (%)
Lung tissue	0.008 (0.007–0.009)	0.037 (0.010–0.044)	2.750 (1.000–7.300)
Tumor tissue	0.040 (0.002–0.100)	0.1000 (0.01–0.390)	3.000 (0.900–24.000)
Fold change	5.0	2.7	1.09

Results are expressed as the median and *P*_25_–*P*_75_ values

Although the difference in the level of EpCAM^+^ cells in relation to tumor histopathology was not significant (*p* = 0.19), we observed a trend toward higher numbers of positive cells in cases of adenocarcinoma, and the median value of EpCAM^+^ cells was 24 % in adenocarcinoma vs. 1.5 % in squamous cell carcinoma.

### Peripheral Blood

To evaluate the specificity of flow cytometry, blood samples from 15 healthy subjects were analyzed. CD133^+^EpCAM^−^ cells were found in all samples as expected (median: 0.0092 %), while CD133^−^EpCAM^+^ and CD133^+^EpCAM^+^ cells were not detected in any healthy donor (data not shown).

Cells expressing CD133 only were detected in 36 (87 %) patients, and cells that were only EpCAM-positive were found in the blood of 25 (60 %) patients. The median values of the proportion and absolute number of CD133^+^ and EpCAM^+^ cells are presented in Table 3. We detected double-positive CD133^+^EpCAM^+^ cells in the blood of 15 (36 %) patients at a median frequency of 0.0004 %.

**Table 3:** Results from the analysis of CD133 and EpCAM expression in the peripheral blood of patients with lung cancer

	All	Metastases	No metastases	Fold change (meta vs. no meta)
CD133^+^EpCAM^−^ (%)	0.0022 (0.0009–0.0036)	0.0021 (0.0011–0.0034)	0.0022 (0.0006–0.0039)	0.95
CD133^+^EpCAM^−^ (per 1 ml)	204 (67–333)	247 (141–405)	138 (48–313)	1.80
CD133^−^EpCAM^+^ (%)	0.0026 (0.0015–0.0063)	0.0015 (0.0004–0.0045)	0.0046 (0.0024–0.0075)	0.32
CD133^−^EpCAM^+^ (per 1 ml)	260 (54–354)	138 (34–303)	298 (213–521)	0.46
CD133^+^EpCAM^+^ (%)	0.0004 (0.0003–0.0008)	0.0004 (0.0002–0.0008)	0.0005 (0.0003–0.0008)	0.8
CD133^+^EpCAM^+^ (per 1 ml)	40 (31–83)	49 (34–132)	39 (15–83)	1.26

Data are shown for all patients and grouped according to metastasis status. Results are expressed as the median and *P*_25_–*P*_75_ values or the percentage of the total cell count

**Fig. 3:**
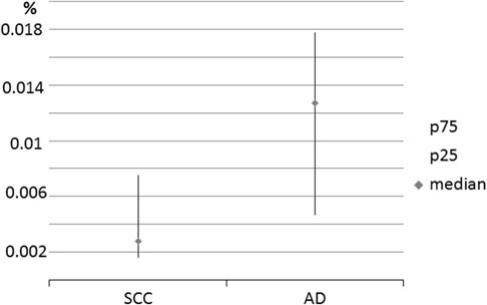
Differences in the proportion of EpCAM^+^ cells in the peripheral blood of lung cancer patients according to tumor histopathological type. *SCC* squamous cell carcinoma, *AD* adenocarcinoma. Data are expressed as the median, *P*_25_–*P*_75_ of the peripheral blood nuclear cells

CD133^+^EpCAM^−^ and CD133^−^EpCAM^+^ cells were found in NSCLC and in SCLC tissues in similar proportions. However, the absolute number of CD133^+^EpCAM^−^ cells was higher in patients with SCLC than NSCLC (299 vs. 204 cells/ml, *p* = 0.19). We also found that the fraction of CD133^−^EpCAM^+^ cells varied between the histopathological types of non-small cell lung cancer evaluated, and this fraction was increased in the blood of patients with adenocarcinoma as compared to squamous cell carcinoma (0.0130 vs. 0.0027 %, *p* = 0.2; Fig. 3).

**Fig. 4:**
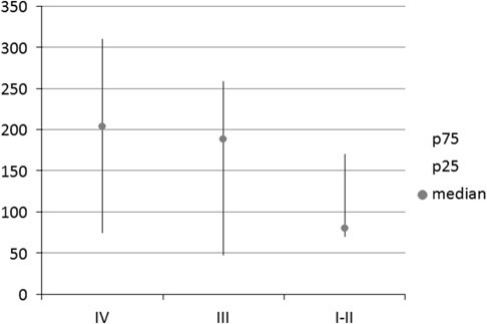
Number of CD133^+^EpCAM^−^ cells per ml of peripheral blood from patients with lung cancer in relation to disease stage I, II, III or IV. Data are expressed as the median, *P*_25_–*P*_75_

We next compared the proportion of analyzed cells between patients with advanced disease (i.e., stages IIIB and IV) and less advanced disease (i.e., stages I–IIIA), and found that the proportion of CD133^−^EpCAM^+^ cells was significantly lower in the blood of patients with advanced disease (0.0018 vs. 0.0067; *p* = 0.018). Additionally, the number of CD133^+^EpCAM^−^ cells per ml of blood varied in relation to disease stage, and the highest number of CD133^+^EpCAM^−^ cells was found in the blood of patients with stage IV disease (Fig. 4).

**Fig. 5:**
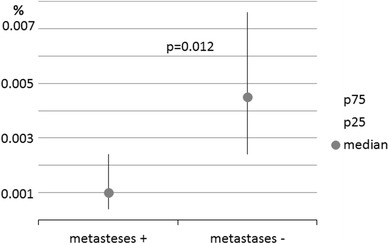
Proportion of EpCAM-positive cells as the percentage of peripheral blood nuclear cells count in patients with lung cancer with or without distal metastases. Data are expressed as the median, *P*_25_–*P*_75_

The proportion and absolute number of cells expressing CD133 or EpCAM only and the combination of both markers, detected in the blood of patients with lung cancer in relation to distal metastases are shown in Table 3. We found an elevated proportion and number of CD133^+^ cells in the blood of patients with metastatic disease, while cells expressing EpCAM were significantly depleted in patients with metastases (median value: 0.0015 vs. 0.0046 % in patients without metastases; *p* = 0.012; Fig. 5). Interestingly, the number of CD133^+^EpCAM^+^ double-positive cells per 1 ml of blood was higher in patients with metastatic disease, although this result was observed in only three patients.

Additionally, we observed the presence of SSEA-4^+^ cells in 67 % of the patients examined at a median expression level of 0.00011 % (100/ml). Interestingly, there was a significant correlation (*p* < 0.05, *R* = 0.64) between the percentage of SSEA-4^+^ cells and the percentage of EpCAM^+^ cells.

## Discussion

The concept of CSCs has shed new light on tumor biology. CSCs, which are thought to possess unique capacities such as giving rise to an entire heterogeneous tumor mass and surviving therapeutic regimens, are candidate metastasis-initiating cells. Eramo et al. (2008) and Tirino et al. (2009) identified a lung CSC subpopulation among CD133-expressing cancer cells; moreover, these cells were shown to be capable of generating tumor xenografts, acquiring specific linear markers upon differentiation and escaping chemotherapy-induced apoptosis. Because CSCs and normal stem cells show similar trafficking abilities in response to the CXCR4/SDF-1 axis (Kucia et al. 2005), we hypothesized that lung CSCs would egress into the circulation. This study sought to investigate whether these cells could be found in the peripheral blood of patients with lung cancer and therefore contribute to the development of metastasis.

We first investigated whether cells with a putative LCSC phenotype could be isolated from freshly obtained tumors. To identify these cells, we chose to stain for the CD133 and EpCAM molecules, as these double-positive cells were previously shown to possess stem cell characteristics in lung cancer (Eramo et al. 2008). The CD133 antigen is commonly used to identify normal and CSCs, and anti-EpCAM staining was performed in this study because this molecule is often overexpressed in lung cancers, although its functional role in forming metastases remains controversial (van der Gun et al. 2010). We confirmed the presence of CD133^+^EpCAM^+^ cells in lung tumor tissues, and these cells were also found in the normal lung parenchyma but at a much lower frequency. We also noted a tendency toward higher EpCAM expression in cases of adenocarcinoma as compared to squamous cell carcinoma, which is consistent with previous data (Went et al. 2006).

The presence of CD133^+^EpCAM^+^ cells in the normal tissue and their higher number in the tumor suggest that cells of such phenotype are important for the development of cancer and these particular cells may be the target of carcinogenesis, but this hypothesis needs further research. However, we can explain this interesting result by the fact that in “healthy” lung the process of carcinogenesis is also possible, taking into account the individual genetic predisposition and influence of environmental factors. Immunohistochemical comparison of the CD133^+^ cells on normal and malignant tissue could be useful to clarify the results obtained by flow cytometry. Also, the functional assays were not the objective of this clinical observation study.

To evaluate the presence of circulating CSCs, we utilized flow cytometry. Although several techniques can be used for the identification of circulating cancer cells, such as PCR, microchips, enrichment and cytological evaluation, flow cytometry was selected for the current study. With flow cytometry, numerous parameters can be simultaneously recorded, including cell morphology, selected marker expression, and marker co-expression. These properties enable the exclusion of cellular debris, dead cells and free circulating mRNA, which can generate artifacts and false-positive errors. Taken together, the analysis of surface protein expression using flow cytometry has been shown to be a relevant method, as CTCs have been detected in patients with lung cancer (based mostly on cytokeratin expression) by numerous groups (Devriese et al. 2012; Reckamp et al. 2009). Although some CTC pre-enrichment techniques (CellSearch, isolation by size of epithelial tumor cells) are often used, we chose to apply standard flow cytometry for our purpose. The choice of flow cytometry was based on its advantages over PCR-based methods, as it enables the colocalization of analyzed markers on the single cell level. On the other hand, during the pre-enrichment steps some cell subpopulations are lost, which limits the variety of data obtained for analyses of correlations. Our preliminary results revealed that EpCAM-positive cells could be identified in the blood of cancer patients with this method, while no EpCAM^+^ or CD133^+^EpCAM^+^ cells could be found in healthy controls. Also, flow cytometry proved its utility to enumerate CTC in other tumors (Rao et al. 2005; Wang et al. 2012). However, the current study is the first report in which circulating LCSCs were analyzed. The same immunocytochemical staining strategy was applied to tumor tissues, and we phenotypically identified CD133^+^EpCAM^+^ CSCs in 36 % of the blood samples evaluated. Although most of the patients studied were in an advanced disease stage, CSCs were absent in only a few cases. This result is not surprising, as circulating cancer cells were found in 46 % of patients in a recent study by Devriese et al. (2012), which suggests that circulating CSCs would be identified at an even lower frequency. Additionally, we did not find any relationship between disease stage and the presence and number of CD133^+^EpCAM^+^ cells. This finding may be related to the limited number of patients and the few cases of non-advanced cancer studied. Moreover, a study by Theodoropoulos et al. (2010), which identified circulating putative breast CSCs, also failed to find clinical correlations with CSCs.

An additional significant finding of the current study concerns the identification of circulating cells expressing the EpCAM antigen alone. As mentioned previously, this molecule is often overexpressed in lung cancers and has been used to detect and enrich circulating cancer cells (Devriese et al. 2012). CD133^−^EpCAM^+^ cells were found in the blood of more than half of the patients studied, and we observed that the percentage of CD133^−^EpCAM^+^ cells in the peripheral blood varied according to histological tumor type. Patients with adenocarcinoma had a higher number of these cells than patients with squamous cell carcinoma, and this finding corresponds to previous results observed in tumor tissues (Baeuerle and Gires 2007; Theodoropoulos et al. 2010; Went et al. 2006). To the best of our knowledge, these differences in the circulating EpCAM^+^ cells in the context of tumor histopathology have not been previously reported. Moreover, in our study population, the proportion of EpCAM-positive cells was higher in patients without distal metastases, which may have some clinical implications. The significance of the presence and identification of EpCAM in cancers depends on the cancer type (van der Gun et al. 2010). In renal clear cell carcinoma and thyroid carcinoma, EpCAM expression has been correlated with improved survival, and in lung cancer, EpCAM overexpression was also associated with improved (Baeuerle and Gires 2007) or not worse prognosis (Kim et al. 2009). The mechanism of action of this molecule is pleiotropic, and EpCAM has been described as a highly immunogenic tumor-associated antigen (Baeuerle and Gires 2007). A possible explanation of this observation is that the expression of EpCAM is related to more differentiated status of CTCs.

SSEA-4 is an embryonic marker that, in adults, can be found in primitive stem cells, such as small embryonic-like stem cells (Kucia et al. 2007), or certain types of tumor cells, as recently reported for epithelial ovarian carcinoma (Ye et al. 2010). In our study, the proportion of SSEA-4-positive cells correlated with cells expressing EpCAM. However, a small number of cases were evaluated, and the origin of the SSEA-4-positive circulating cells in patients with lung cancer requires further study.

We also analyzed the CD133^+^EpCAM^+^ cells in the peripheral blood of patients with lung cancer. This subpopulation was found at various levels in almost all of the samples examined. The absolute number of these cells was higher in patients with distal metastases, and this number positively correlated with the clinical lung cancer stage (i.e., greatest number found among patients with stage IV disease). The CD133 molecule is expressed by numerous stem cells, although it is mostly expressed by hematopoietic stem cells and endothelial progenitor cells in the peripheral blood (Dome et al. 2006; Vroling et al. 2010). An elevated number of endothelial progenitor cells (CD133^+^CD34^+^VEGFR2^+^) in patients with lung cancer have been previously reported, which was shown to correlate with disease stage and clinical behavior (Dome et al. 2006). Recently, hematopoietic stem cells (identified as CD133^+^CD34^+^CD45^+^ cells) were also found in the peripheral blood of patients with lung cancer, and their number negatively correlated with time to progression (Vroling et al. 2010). According to these studies, we would predict that CD133^+^ cells are adult stem cells and mostly hematopoietic and endothelial in nature. Therefore, the current study identified the potential utility of measuring CD133 expression as a potential biomarker in patients with cancer.

The results of our study seem to corroborate the cancer stem cell theory and the presence maturation continuum among both normal and cancer cells. Primitive progenitor cells expressing CD133 and EpCAM proteins are rare, but they expand in lung cancer compared to the healthy lung. Cells expressing EpCAM antigen only have differentiated phenotype and their frequency is almost equal in the normal and malignant tissue. Analysis of peripheral blood led us to a similar conclusion, as CTCs of patients without distal metastases had differentiated CD133^−^EpCAM^−^ phenotype.

Aside from several strengths, our study had some limitations. First, we used only one technique to analyze putative CSCs and, therefore, further research is warranted. The blood volume used in our study is low, but we still managed to identify EpCAM and CD133-positive cells and clinical differences correlating to their frequencies. Additionally, this study did not find any significant correlation between the number of circulating CD133^+^EpCAM^+^ cells and the clinical data. One potential reason for this inconclusive result may have been the limited number of cases studied. Unfortunately, we were unable to study and analyze stem cell markers in tumors and blood from the same patients. Additionally, the patient group included NSCLC and SCLC patients, and most of these individuals had been diagnosed with advanced disease.

In conclusion, this study confirmed the existence of CD133^+^EpCAM^+^ cells in lung tumor tissues and, for the first time, identified these cells in the peripheral blood of patients with lung cancer. Additionally, we demonstrated that the number of circulating EpCAM-positive cells varied depending on the lung tumor type and the presence of distal metastases. Cells expressing the CD133 antigen alone, which are likely hematopoietic and endothelial progenitors, were shown to correlate with the lung cancer stage and could potentially be useful as a biomarker of tumor spread.
